# Fitting mechanistic epidemic models to data: A comparison of simple Markov chain Monte Carlo approaches

**DOI:** 10.1177/0962280217747054

**Published:** 2018-05-30

**Authors:** Michael Li, Jonathan Dushoff, Benjamin M Bolker

**Affiliations:** 1 Department of Biology, McMaster University, Hamilton, Ontario, Canada; 2 Department of Mathematics and Statistics, McMaster University, Hamilton, Ontario, Canada; 3 Institute for Infectious Disease Research, McMaster University, Hamilton, Ontario, Canada

**Keywords:** Markov chain Monte Carlo, Hamiltonian Monte Carlo, discrete-time susceptible-infectious-removed model, dispersion, moment-matching

## Abstract

Simple mechanistic epidemic models are widely used for forecasting and parameter estimation of infectious diseases based on noisy case reporting data. Despite the widespread application of models to emerging infectious diseases, we know little about the comparative performance of standard computational-statistical frameworks in these contexts. Here we build a simple stochastic, discrete-time, discrete-state epidemic model with both process and observation error and use it to characterize the effectiveness of different flavours of Bayesian Markov chain Monte Carlo (MCMC) techniques. We use fits to simulated data, where parameters (and future behaviour) are known, to explore the limitations of different platforms and quantify parameter estimation accuracy, forecasting accuracy, and computational efficiency across combinations of modeling decisions (e.g. discrete vs. continuous latent states, levels of stochasticity) and computational platforms (JAGS, NIMBLE, Stan).

## 1 Introduction

Simple homogeneous population models have been widely used to study emerging infectious disease outbreaks. Although such models can provide important insights – including estimated epidemic sizes and predicted effects of intervention strategies, as well as short-term forecasts – they neglect important spatial, individual-level and other heterogeneities. Decades of work have created frameworks that enable researchers to construct models that capture many of these more realistic aspects of infectious disease epidemics. But many challenges remain. In particular, estimating parameters (and associated uncertainties) is always challenging, especially for models incorporating multiple forms of heterogeneity, and especially during the early stages of an epidemic when data are limited. Using complex models that are insufficiently supported by data can lead to imprecise and unstable parameter estimates^1^ – in such cases, researchers often revert to simpler models for practical purposes.

In the past few decades, researchers have begun to adopt Bayesian approaches to disease modeling problems. Bayesian Markov Chain Monte Carlo (MCMC) is a powerful, widely used sampling-based estimation approach. Despite the widespread use of MCMC in epidemic modeling,^2,3^ however, there have been relatively few systematic studies of the comparative performance of statistical frameworks for disease modeling.^4^

In this paper, we apply relatively simple MCMC approaches to data from simulated epidemics that incorporate stochasticity in both transmission and observation, as well as variable generation-interval distributions (not assumed to be known when fitting). We compare model approaches of varying complexity, including an estimation model that matches the simulation model. For each model, we quantify parameter estimation accuracy and forecasting accuracy; this sheds light on which phenomena are most important to include in models to be used for estimation and forecasting.

We also compare three different MCMC platforms: JAGS,^5^ NIMBLE^6^ and Stan.^7^ In principle, for any given model, any valid method of MCMC sampling should eventually converge on the same (correct) posterior distribution. However, even with the relatively simple models considered here, a theoretically valid software package can experience problems in practice: we wanted to investigate this phenomenon. Furthermore, even when different platforms converge to essentially the same result, they may show large differences in computational efficiency: we therefore also quantify efficiency for the models we study.

## 2 Methods

We generated test data using a simple framework that combines a *transmission process* based on a simple discrete-time model with an *observation process* to account for incomplete reporting. Both processes are assumed to be stochastic. We then fit the observed cases from these simulations using Bayesian methods that model the underlying true number of infections as a latent (i.e. unobserved) variable. Our Bayesian fitting models explore an approach that matches the assumptions of the simulation model, as well as various simplifications: in particular, we explore simpler methods of accounting for variation in both the transmission process and the observation process, and the use of continuous rather than discrete latent variables. For simplicity, we have here assumed that data are reported on the same discrete time scale on which the disease process is simulated (but not that the reporting period is the same as the generation time of the disease; see below). This assumption requires that the generation time be at least as long as the reporting period. It would be relatively straightforward to relax this assumption, for example by assuming that the epidemic dynamics occur on a finer time scale than the reporting interval, or by simulating in continuous time but fitting with a discrete-time model; we do not explore these questions here.

### 2.1 Simulation model

The transmission process of our dual-process framework is based on the Reed-Frost chain binomial model, which can also be described as a discrete-time, stochastic compartmental SIR model.^8^ To account for the possibility that some fraction of the population may be beyond the scope of the epidemic – geographically or socially isolated, genetically resistant, vaccinated or immune due to previous exposure – we assume that only a proportion *P*_eff_ of the total census population is actually susceptible to infection. We further assume that, in every time step, only a proportion (randomly chosen with mean *P*_rep_) of new infections are actually observed. We model both transmission and observation using a beta-binomial (rather than binomial) distribution to account for additional sources of variation (i.e. overdispersion) in both processes. The equations are

(1) $$\begin{array}{c} N_{\text{eff}}=P_{\text{eff}}N \end{array}$$

(2) $$\begin{array}{c} S_{1}=N_{\text{eff}}-I_{1} \end{array}$$

(3) Φt= ∑i=1ℓk(i)It−ℓ+i

(4) $$\begin{array}{c} I_{t+1}\;∼\text{Beta Bin}(1-e^{-\Phi_{t}},S_{t},\delta_{P}) \end{array}$$

(5) $$\begin{array}{c} S_{t+1}=S_{t}-I_{t+1} \end{array}$$

(6) $$\begin{array}{c} {\text{Obs}}_{t}\;∼\text{Beta Bin}(P_{\text{rep}},I_{t},\delta_{\text{obs}}) \end{array}$$

where $\Phi_{t}$ is the force of infection at time *t*; $N_{\text{eff}}$ is the effective population size; and ℓ is the number of lags.

**Figure 1.**
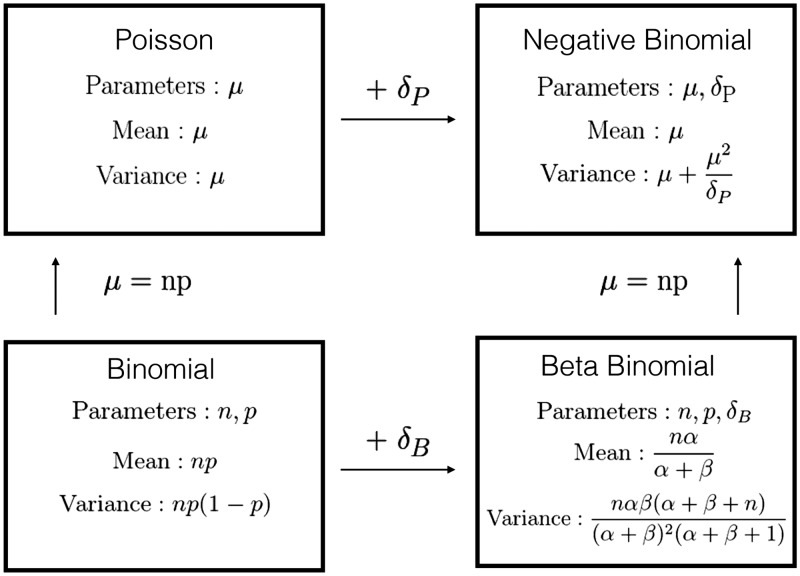
Discrete distribution relationships. For beta-binomial distribution (bottom right panel), we used an alternative parameterization *α* and *β*, where *α* = $\frac{\delta_{B}}{1-p}$ and *β* = $\frac{\delta_{B}}{p}$. Moving from the top to bottom row adds a size parameter (replacing *μ* with *np*). Moving from left to right adds a dispersion parameter *δ_P_* and *δ_B_* for Poisson and Binomial distribution, respectively.

The most common parameterization of the beta-binomial comprises three parameters: the binomial size parameter *N* plus two additional shape parameters (*α* and *β*) that describe the Beta distribution of the per-trial probability. Uses of the beta-binomial in statistical modeling instead typically transform the shape parameters into a pair of parameters that describe the per-trial probability and a dispersion parameter^9^; larger values of the dispersion parameter *δ* correspond to less variability. We use a slight modification of this parameterization (see Figure 1).

We extend the Reed-Frost model by allowing the infectious period to last longer than one step, and the infectivity to vary based on how long an individual has been infected; we do this by parameterizing a transmission kernel that describes the force of infection coming from individuals who were infected ℓ time steps ago. For convenience, we assumed a fixed maximum window length (ℓ=5). We then based our transmission kernel on a negative binomial distribution, truncated to fit this window:

(7) $$\begin{array}{c} \overset{∼}{k}(i)=i^{\left(G_{S}-1\right)}\times \text{exp}(\frac{-i}{G_{P}\times \ell }),i=1,\ldots ,\ell \end{array}$$

(8) $$\begin{array}{c} k(i)=\frac{R_{0}}{N_{\text{eff}}}\times \frac{\overset{∼}{k}(i)}{\sum_{i=1}^{\ell }\overset{∼}{k}(i)},i=1,\ldots ,\ell \end{array}$$

Here, $R_{0}$ represents the basic reproductive number and *G_S_* and *G_P_* are shape and position parameters, respectively.

### 2.2 Fitting model

#### 2.2.1 Transmission and observational process errors

The transmission (equation(4)) and observation (equation (6)) processes in the simulation model are both defined as beta-binomial (BB) processes. In fitting, we used the BB to match the simulation model, but also tried several simpler alternatives: binomial (B), Poisson (P), and negative-binomial (NB) processes. Process B does not allow for overdispersion, while NB does not incorporate the size of the pool from which a value is chosen; that is, it is theoretically possible for a NB sample of the number of infections to be larger than the current susceptible population (although this is extremely unlikely when the *per capita* infection probability is small). Process P neglects both of these phenomena. Figure 1 illustrates the relationship of the four discrete distributions.

#### 2.2.2 Multiple scale decorrelation

The proportion of the population assumed to be effectively susceptible (*P*_eff_) and the reporting proportion (*P*_rep_) has very similar effects on observed incidence. We therefore reparameterized the model so that it uses a single parameter *P*_effrep_ for their product, and a second to govern how the product is apportioned between the two quantities:

(9) P^eff=Peffrep1-ρ

(10) P^rep=Peffrepρ

We expected a priori that this parameterization would improve statistical convergence, since it makes it possible to sample different values of the poorly constrained value of *ρ* without changing *P*_effrep._ It is straightforward to back-calculate *P*_eff_ and *P*_rep_ once the model is fitted. For similar reasons, we experimented with measuring infected individuals on a “reporting” scale in our continuous-variable models (see below).

#### 2.2.3 Continuous latent variables

**Figure 2.**
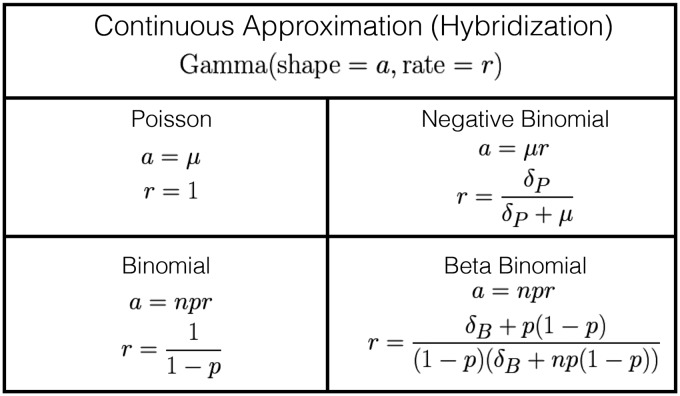
Continuous approximation of discrete distributions via moment matching. Distributions in Figure 1 were matched to a Gamma distribution with equivalent first and second moments.

Another simplification we considered was treating the unobserved number of underlying cases as a continuous variable. To do this, we matched the first two moments of the discrete distribution to a Gamma distribution (Figure 2).

Equations (4) and (6) can be rewritten as

(11) I^t+1 ∼Gamma(a,rPrep)

(12) Obst ∼NB(It^,δobs)

One advantage of this continuous approximation approach is that it allows us to scale our latent variable to help with model convergence, so that infected individuals are measured on the reporting scale. Another advantage is that it allows us to use Hamiltonian Monte Carlo (HMC), which cannot easily use discrete latent variables.

### 2.3 Bayesian Markov Chain Monte Carlo

In Bayesian MCMC, model parameters are sampled from the posterior distribution by a reversible Markov chain whose stationary distribution is the target posterior distribution. Classical MCMC techniques include the Metropolis-Hasting algorithm,^10^ Gibbs sampling,^11^ and slice sampling.^12^ Recently, convenient implementations of a powerful MCMC technique called Hamiltonian Monte Carlo (HMC: also called hybrid MC)^13^ have become available. HMC uses the concept of Hamiltonian dynamics to create a proposal distribution for the M-H algorithm, together with the leap-frog algorithm and the No U-Turn sampler.^14^ HMC requires more computational effort per sample step compared to other MCMC techniques, but because subsequent steps are less strongly correlated it also produces more effective samples per sample step.^7,14^

#### 2.3.1 Platforms

Many software platforms implement the automatic construction of MCMC samplers for user-defined models. One of the most widely used platforms is JAGS (Just Another Gibbs Sampler); despite its name, it implements a variety of MCMC techniques to fit models. NIMBLE (Numerical Inference for Statistical Models for Bayesian and Likelihood Estimation) is a more recent platform that allows users to flexibly model and customize different algorithms and sampling techniques for MCMC. Neither JAGS nor NIMBLE has yet implemented HMC. One of the relatively few platforms that currently implements HMC is Stan, which provides full Bayesian inference for continuous-variable models based on the No-U-Turn sampler, an adaptive form of HMC.

#### 2.3.2 Simulation and evaluations

We evaluated our estimates of (1) total cases predicted over the forecast window (disaggregated forecasts are analyzed in the supplementary material) and (2) key model parameters, including the estimated mean generation interval (MGI:defined as∑i=1ℓik^(i)∑i=1ℓk^(i)). We used bias, root mean square error (RMSE), and coverage to assess model fit. Bias and RMSE are based on proportional errors, defined as the log ratio of our estimate (taken as the median of the posterior sample) to the known true value from our simulations. Errors were compared on the log scale in order to allow comparison of the accuracy of estimation of different parameters that may be on very different scales. The median is a scale-invariant, robust summary statistic for the location parameter of a Bayesian posterior distribution.^15^ Thus in order to compare different parameters in a consistent, unitless fashion, the errors were calculated as εi=log(med(θi^)/θi). We then calculated bias (median(ε)) and RMSE ($\sqrt{\text{mean}(\varepsilon_{i}^{2})}$).

Coverage refers to the frequency with which the computed confidence intervals include the true values of parameters or simulated quantities such as the forecast number of cases. We used 90% quantile-based intervals to evaluate coverage (i.e. a range from the 0.05 to the 0.95 quantile of the sampled posterior distributions).^16^

Evaluating the coverage of Bayesian model estimates based on simulated parameters runs the risk of confounding two questions: How well does the modeling implementation work? and How appropriate are the prior distributions for the particular question? In particular, when tested parameters are from regions with high prior density, coverage is biased upwards (i.e. it will be higher than the nominal value when the method is working properly) – particularly problematic is that this bias may make fits look good when in fact they are under-covering. This scenario can easily occur if we follow the standard frequentist simulation scheme of simulating all epidemic realizations with the same fixed set of parameters, then choose Bayesian priors that are centered on or near the fixed parameters. One potential solution is to use only uninformative priors (so that the simulation parameter values do not have high prior density); this was both impractical, because completely uninformative priors led to numerical instability in our fitting procedures, and unrealistic, because it is likely that researchers would use informative priors in a real epidemic-fitting exercise.

As an alternative way to resolve this situation, we implemented an established Bayesian validation protocol^16^ where we: draw parameters from our assumed prior distribution; generate data using the drawn parameters; and fit the Bayesian model with the same prior distributions. This scheme matches the assumptions of our model, and is therefore a fair way to evaluate how well the implementation works. We sampled 100 sets of the parameters from the same prior distribution that was used in the fitting process; for each parameter set, we simulated one realization of 15 time steps (10 for fitting and 5 to compare to forecasts). All model variants were used to fit each realization (Tables 1 and 2 in the online appendix give more detail about parameters and priors). We combined two convergence criteria to assess convergence for the main parameters ($R_{0}$, *P*_eff,_ *P*_rep_): we required a value of the Gelman and Rubin statistic R^<1.1 and an effective sample size (ESS) greater than 400 for each replication. For each replication we sample four chains starting with 4000 iterations; we repeatedly double the number of iterations (with a upper threshold of one million iterations) until the convergence criteria are met. Forecasts were made by simulating incidence five time steps forward using parameters sampled from the fitted posterior distributions.

## 3 Results

**Figure 3.**
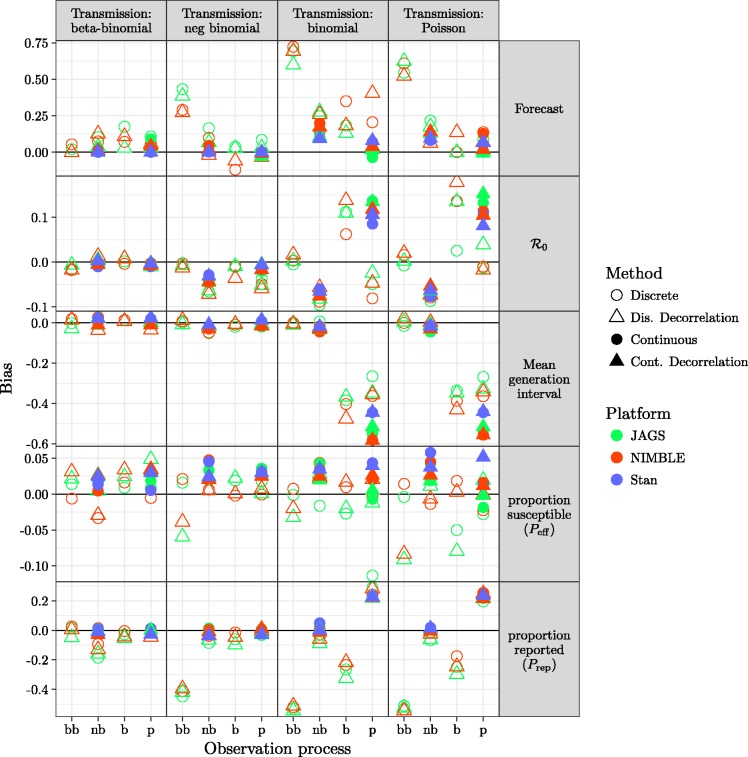
Comparison of bias (based on proportional errors) for forecasts and parameters using models described in section 2.2 across different platforms described in section 2.3.1. Models with overdispersion in the transmission process (BB and NB, leftmost and second-left columns of panels) and models with overdispersion in the observation process (BB and NB, leftmost and second-left x-axis ticks within each panel) have generally low bias. Continuous latent-state models (solid points) are only implemented for negative binomial and Poisson observational processes.

**Figure 4.**
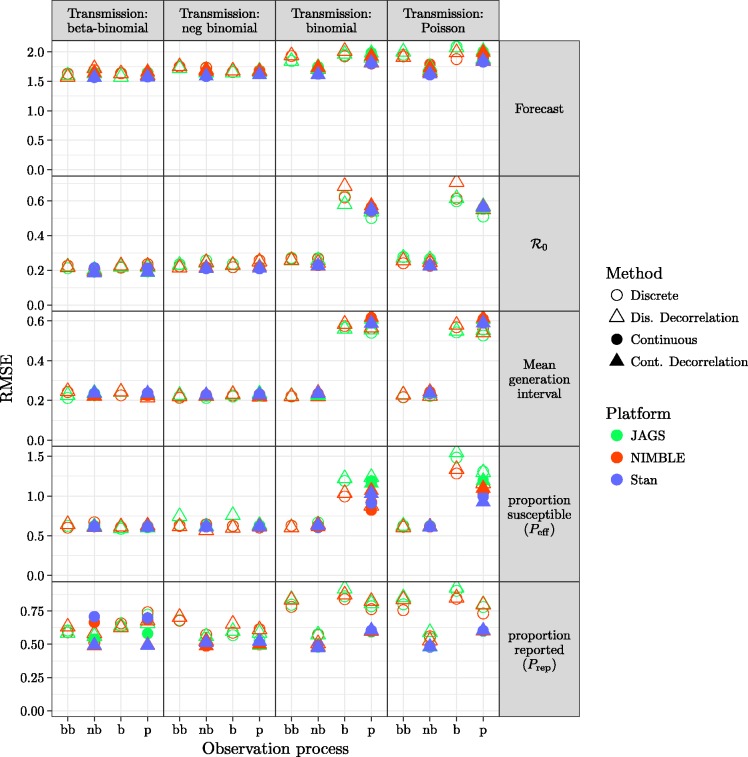
Comparison of RMSE (based on proportional errors) for all fitting model variants. The layout matches that of Figure 3. Patterns across models and platforms are similar to those seen in Figure 3. Short-term forecasts have generally high error, even when bias is low, reflecting inherent uncertainty in the system. The highly correlated parameters *P*_eff_ and *P*_rep_ also show high error but not high bias.

The full model (which matches the simulation model) provides generally good forecasts and parameter estimates as assessed by bias (Figure 3) or RMSE (Figure 4), except for estimates of *P*_eff_ using JAGS.

In general, models with any kind of dispersion in the transmission process, or with negative binomial dispersion in the observation process, did well. The exception is that models that combined negative binomial transmission dispersal with beta binomial observation dispersal produced biased forecasts and estimates of *P*_rep._

There are no clear differences in the quality of model fit due to multi-scale decorrelation, latent continuous transmission process or platform.

**Figure 5.**
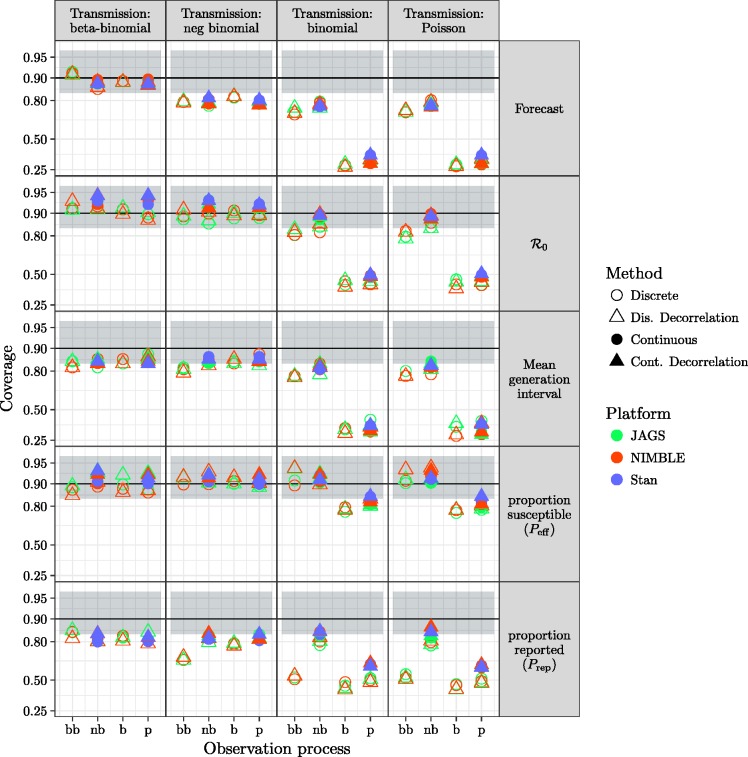
Comparison of coverage probability for forecast and parameters. Models with overdispersion in the transmission process (BB and NB, leftmost and second-left columns of panels) and models with overdispersion in the observation process (BB and NB, leftmost and second-left x-axis ticks within each panel) have coverage near the nominal value of 0.9 for all parameters and model variants. The black line shows the nominal coverage, and the grey ribbon the 95% binomial confidence interval based on 100 simulated fits. Vertical axis is plotted on a logit scale.

Figure 5 shows the statistical coverage of our estimates. Similar to the results for bias and RMSE (Figures 3 and 4), we find generally good coverage (i.e. close to the nominal value of 0.9) for models with dispersion in the transmission process, except that the negative-binomial transmission process model undercovers across the board (coverage ≈0.8 for all observation process models and platforms) for forecasts and *P*_rep._ For models without dispersion in transmission, models with dispersion in the observation process have low coverage (≈0.8) for most parameters, while the beta-binomial process model has low coverage (≈0.4) for *P*_rep_ and models without any dispersion have uniformly low coverage.

**Figure 6.**
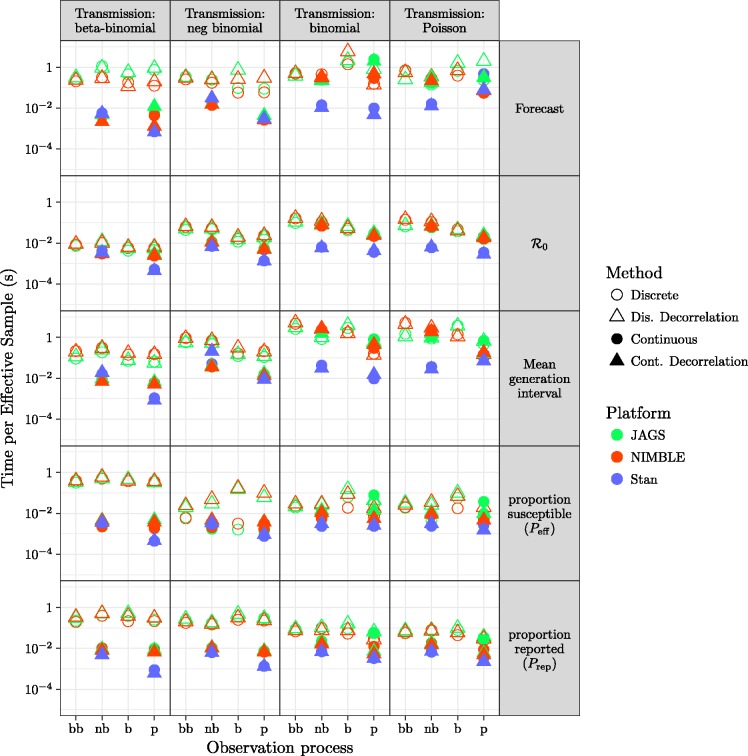
Comparison of efficiency for all fitting model variants: layout of models and platforms as in Figure 3.

There are substantial efficiency differences between transmission-process approaches (continuous vs. discrete), as measured by time per effective sample size, shown in Figure 6. For a given platform, models using continuous latent variables are generally more efficient than discrete latent processes. Comparing models with continuous latent variables between platforms (Figure 5, second and fourth column of every panel), Stan (using HMC) is sightly more efficient for majority of the parameters, followed by NIMBLE and JAGS. Furthermore, continuous latent-variable models (especially using HMC in STAN) use fewer iterations (when meeting all convergence criteria described in section 2.3.2) than discrete latent-variable models.

## 4 Discussion

We have fitted models varying in complexity to simulated epidemic data with multiple sources of heterogeneity, using several different platforms. Using models that include some form of overdispersion is necessary for robust fits, but models that include overdispersion only in the transmission process can work as well as or better than the full model. Including overdispersion only in the observation process (if implemented as a negative binomial distribution) also provides relatively robust fits to these data. Simplifying the models by using continuous rather than discrete latent variables increased efficiency with little effect on the quality of the fits.

### 4.1 Ceilings

The effects of using distributions with ceilings (i.e. binomial and beta-binomial distributions) instead of their less realistic counterparts without ceilings (Poisson and negative binomial) were relatively small. In our framework, ceilings only apply in models with discrete latent variables; the primary effect of such ceilings is to reduce variance as probabilities (of infection or of sampling) become large. (Reporting-process models without ceilings also allow for false positives or over-reporting, which may be important in some contexts.)

### 4.2 Overdispersion

Accounting for overdispersion had more impact on our fits than the presence or absence of ceilings. In particular, models with no overdispersion in either process lacked flexibility and tended to be over-confident (that is, they showed low coverage). However, models that account for overdispersion in only one process (either transmission or observation) tended to be reliable for estimating parameters such as $R_{0}$, mean generation interval, and short-term forecasts, particularly when overdispersion was implemented through the negative binomial (a less constrained distribution than the beta binomial). However, parameters that are closely tied to the details of a particular model structure (such as the overdispersion parameters for the observation and transmission processes) must change when the overdispersion model changes, in order to compensate for missing sources of variability.

Several authors^17,18^ suggest that accounting for process as well as observation error in estimates of $R_{0}$ and in forecasts is necessary in order to avoid over-confident estimates. Our exploration does not include any cases where process error is completely absent – even our “dispersion-free” processes incorporate sampling error in the process. However, we find that neglecting overdispersion can still lead to over-confident and unreliable estimates.

### 4.3 Reporting

In classic infectious disease models, reducing reporting rate and reducing the total effective population size have similar effects: reducing the observed size of the epidemic. While we want to make as few assumptions as possible about unobservable aspects of the epidemic, underreporting is of huge practical importance. Additionally, modeling observation error explicitly is required for reliable estimates of uncertainty.^17^ If reporting error is modeled with a ceiling, then underreporting is a necessary component of reporting error (i.e. reporting will be biased downward in the presence of other sources of noise). Allowing overdispersion decouples the variance from the mean of the reporting process (i.e. the extra overdispersion parameter means that the variance is not determined by the mean).

Because reporting rate and effective population size play similar roles in epidemic dynamics, incorporating them both in a model may make their parameter estimates strongly correlated and hence difficult to identify: we may be very uncertain whether low observed epidemic incidence is driven by a small effective population size or a low reporting rate. We have addressed convergence problems arising from this issue by reparameterizing the model (Section 2.2.2). From a conceptual point of view, joint unidentifiability is not necessarily a serious problem, as long as the quantities we are most interested in (such as $R_{0}$) are identifiable. In practice, however, weak identifiability can cause hard-to-detect convergence problems; known-parameter simulations like those implemented here are useful for validation in such cases.

### 4.4 Extensions and alternative approaches

Our analysis covers classical MC (i.e. conditional updating of parameters via conjugate, slice, and Metropolis-Hastings samplers) and HMC approaches. Even within this scope there is additional room for analysis, both in terms of exploring important heterogeneities that we have neglected here (such as spatial, age and social structure), and in improving sampling techniques (e.g. by adjusting the choice of samplers in JAGS or NIMBLE or by redundant parameterization^19^).

More broadly, a plethora of other model-fitting tools is available to researchers, from deterministic optimization tools based on the Laplace approximation^20,21^ to sequential techniques such as iterated filtering and particle MC.^22–25^ These techniques can in principle be combined flexibly with the methods we explore here, e.g. using HMC to sample top-level parameters while implementing a sequential MC technique for the latent states. It will be interesting to see how the single-technique methods here compete with hybrid approaches, and how flexible toolboxes such as NIMBLE will fare against more focused platforms like Stan.

### 4.5 Prior distributions

This paper focuses on evaluating Bayesian methods for fitting and forecasting epidemics. For the purposes of evaluation, we use parameter distributions for simulation that exactly match our Bayesian priors. We are assuming that researchers have a reasonable method of choosing appropriate Bayesian priors; in real applications this will be an important challenge.

## 5 Conclusion

We have presented a comparison of simple MCMC approaches to fit epidemic data. We learned two things about fitting epidemic data. First, modeling different processes with dispersion (BB and NB) is a naive but effective way to add uncertainty in the model; models that neglect such uncertainty are likely to be over-confident and less accurate at forecasting. Second, approximating discrete latent state process with continuous processes can aid efficiency without losing robustness of fit. This allows more efficient fitting in the classic framework (e.g. JAGS and NIMBLE), and also allows us to use the more advanced HMC technique (which we implemented via Stan).

## Supplemental Material

Supplemental material for Fitting mechanistic epidemic models to data: A comparison of simple Markov chain Monte Carlo approaches Click here for additional data file. Supplemental material for Fitting mechanistic epidemic models to data: A comparison of simple Markov chain Monte Carlo approaches by Michael Li, Jonathan Dushoff and Benjamin M Bolker in Statistical Methods in Medical Research

## Supplementary material

In the main text, we present the bias, RMSE, coverage and efficiency plots for aggregated forecast, $R_{0}$, MGI, *P*_eff,_ and *P*_rep._ Here, we present plots showing the other parameters (shape *G_S_* and position *G_P_* of the transmission kernel and process and observation overdispersion parameters *δ_P_* and *δ_obs_*) and disaggregated forecasts (five forecast steps) that are excluded in the main text. We also add some representative plots of the simulated cases and forecast.
