# Supplementary material for: Fitting mechanistic epidemic models to data: A comparison of simple Markov chain Monte Carlo approaches
Source: Stat Methods Med Res. 2018 May 30;27(7):1956–67. doi: 10.1177/0962280217747054 (PMC6027774; doi:10.1177/0962280217747054)
Supplement: Supplemental material for Fitting mechanistic epidemic models to data: A comparison of simple Markov chain Monte Carlo approaches [file Supplemental_material.pdf]

Supplemental material

In the main text, we present the bias, RMSE, coverage and efficiency plots for aggregated forecast, $\mathcal{R}_0$, MGI, P_{eff}, and P_{rep}. Here, we present plots showing the other parameters (shape G_S and position G_P of the transmission kernel and process and observation overdispersion parameters δ_P and δ_{obs}) and disaggregated forecasts (five forecast steps) that are excluded in the main text. We also add some representative plots of the simulated cases and forecast.

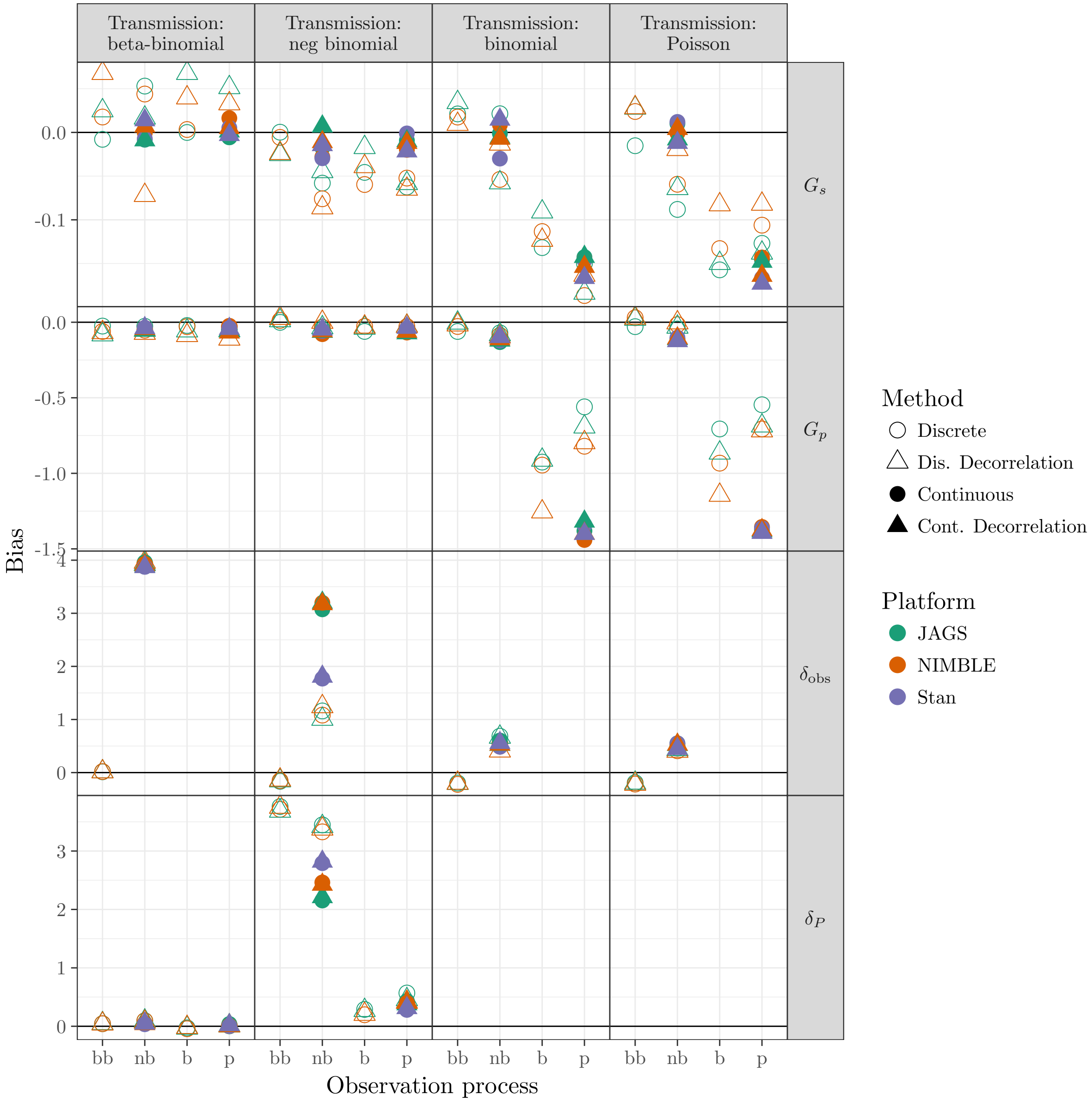

Figure S1. Comparison of bias for G_S (transmission shape), G_P (transmission position), δ_{obs} (observation overdispersion), and δ_P (process overdispersion: more detail given in Sect. 2.2) across different platforms (described in Sect. 2.3.1). Overdispersion parameter δ_P is only applicable in models with dispersion in the transmission process (first and second left column panel) and overdispersion parameter δ_{obs} is only applicable in models with dispersion in the observation process (first and second column within each column panel).

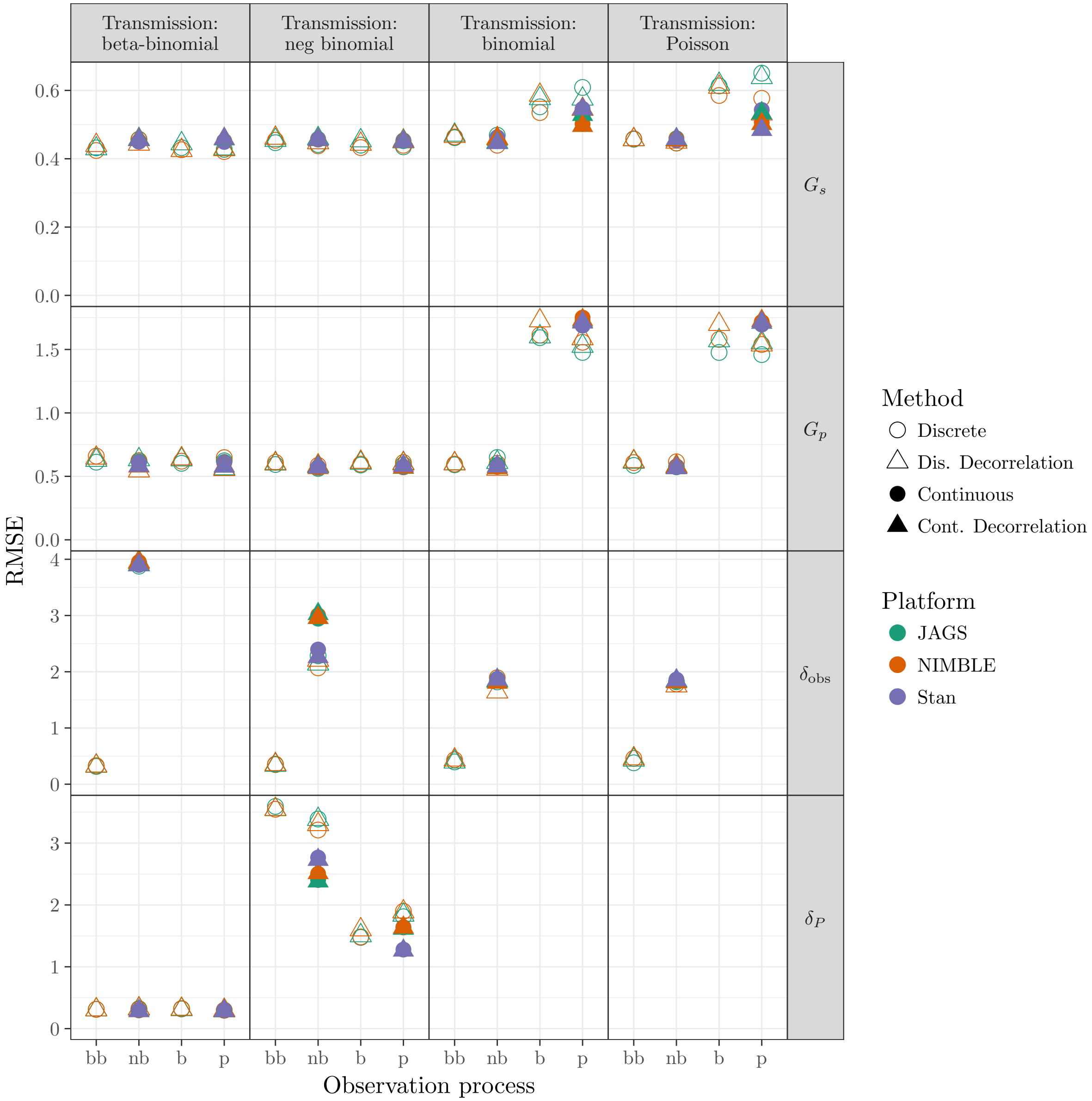

Figure S2. Comparison of RMSE for G_S, G_P, δ_{obs}, and δ_P. See Figure **??** in main text and Figure S1 in appendix for details.

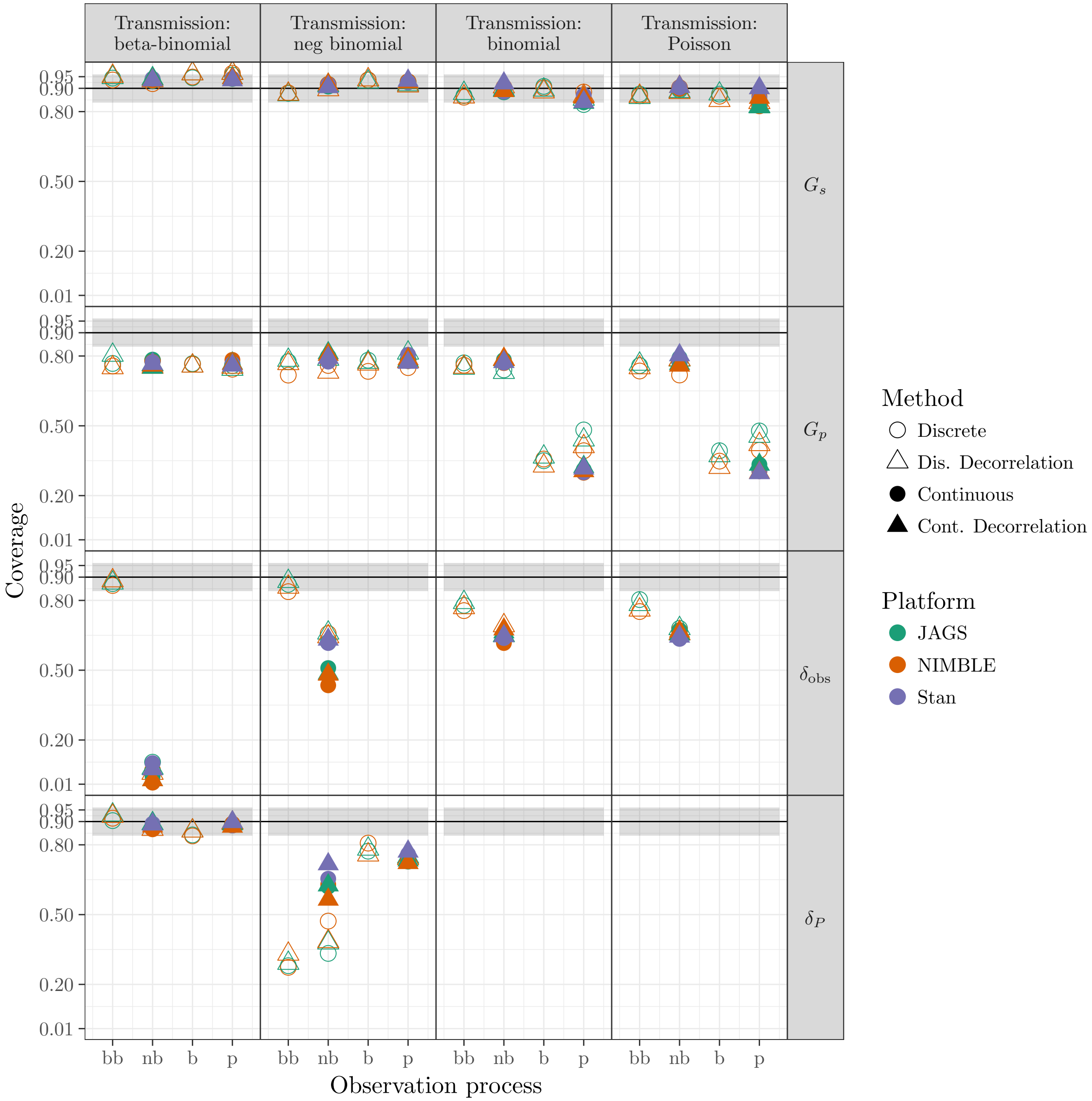

Figure S3. Comparison of coverage for G_S, G_P, δ_{obs}, and δ_P. See Figure **??** in main text and Figure S1 in appendix for details.

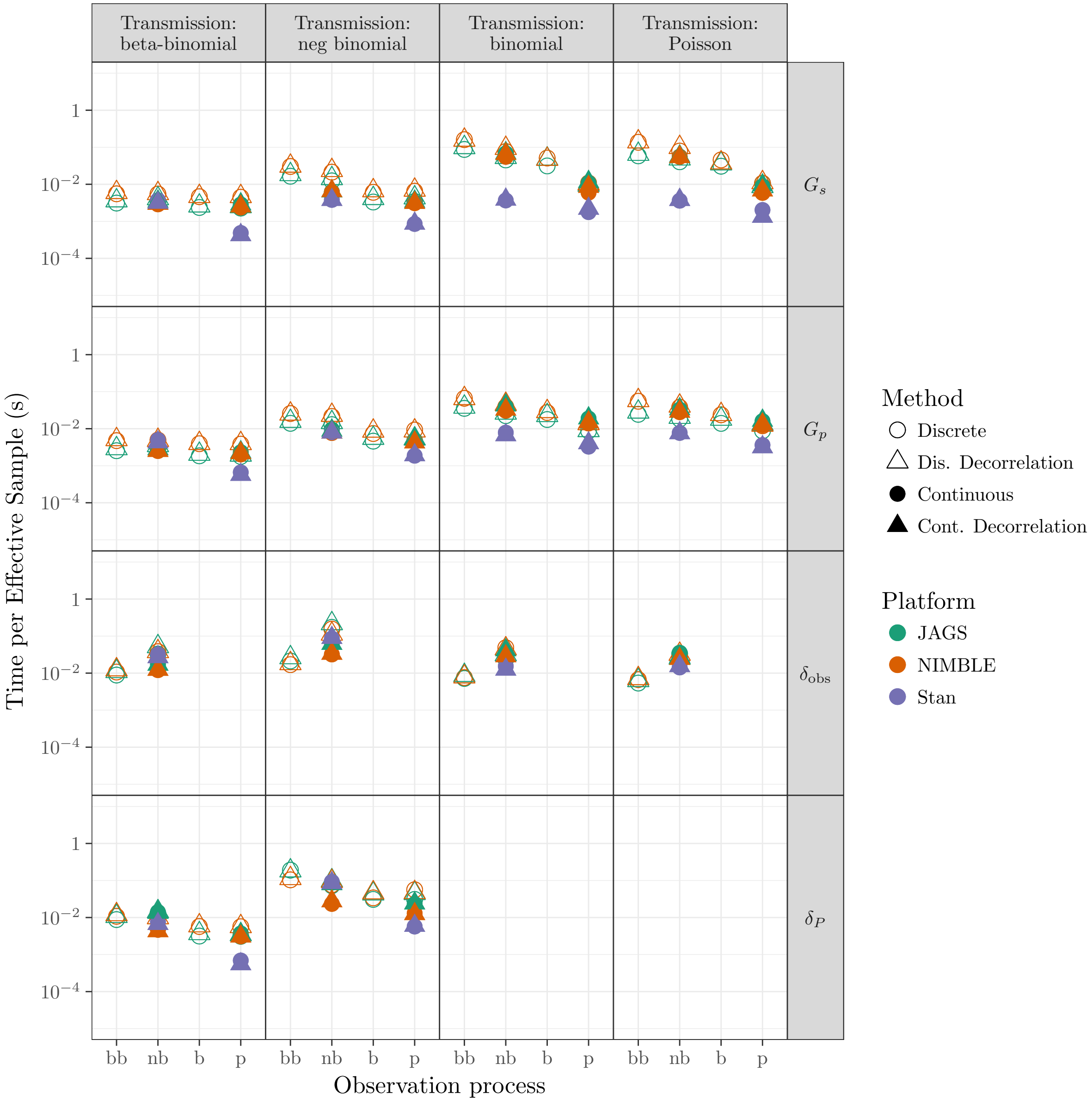

Figure S4. Comparison of coverage for G_S, G_P, δ_{obs}, and δ_P. See Figure **??** in main text and Figure S1 in appendix for details.

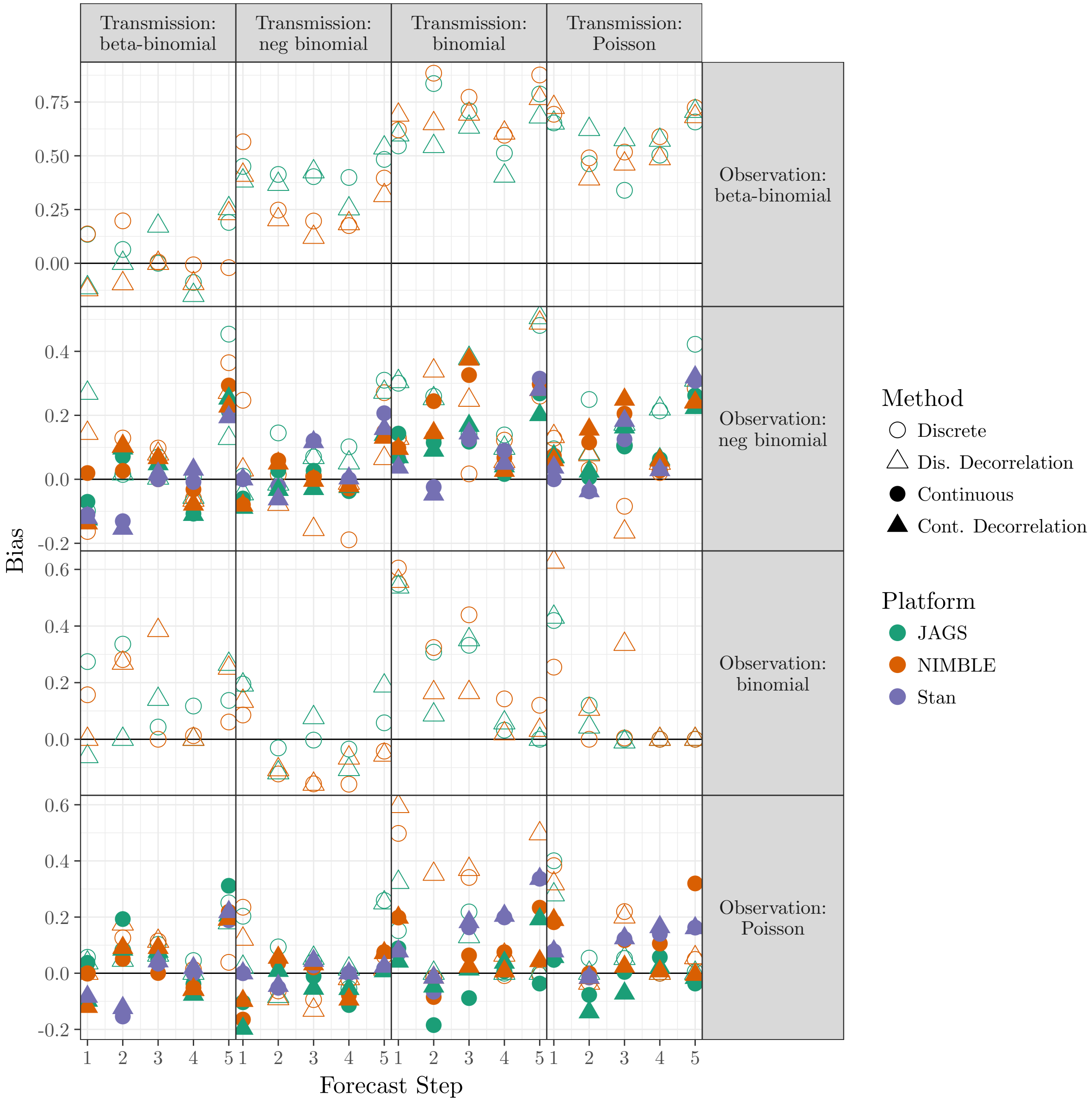

Figure S5. Comparison of bias for five forecast steps (described in Sect. 2.2) across different platforms (described in Sect. 2.3.1).

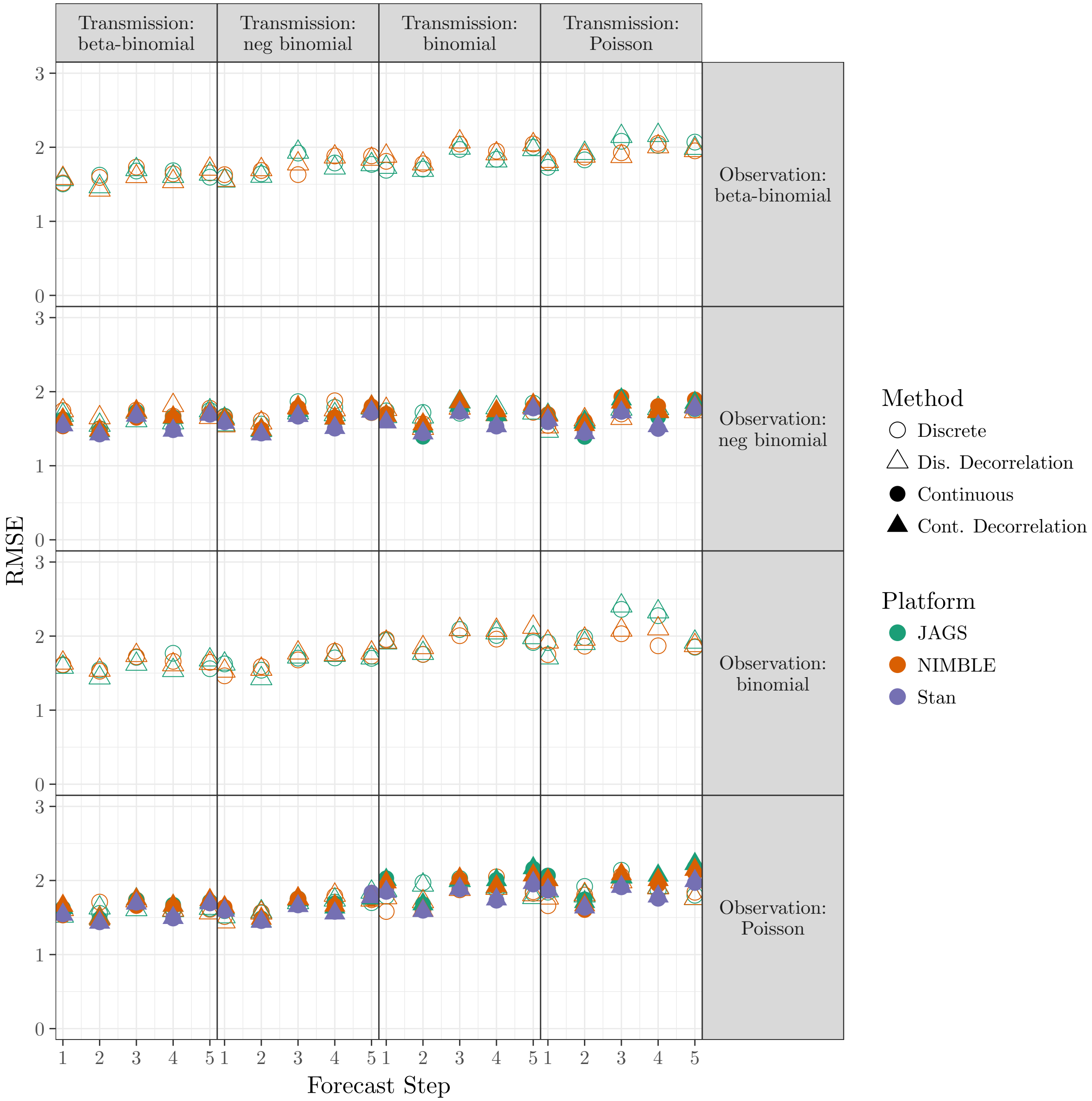

Figure S6. Comparison of RMSE for five forecast steps described in Sect. 2.2 across different platforms described in Sect. 2.3.1. See Figure **??** in the main text for details.

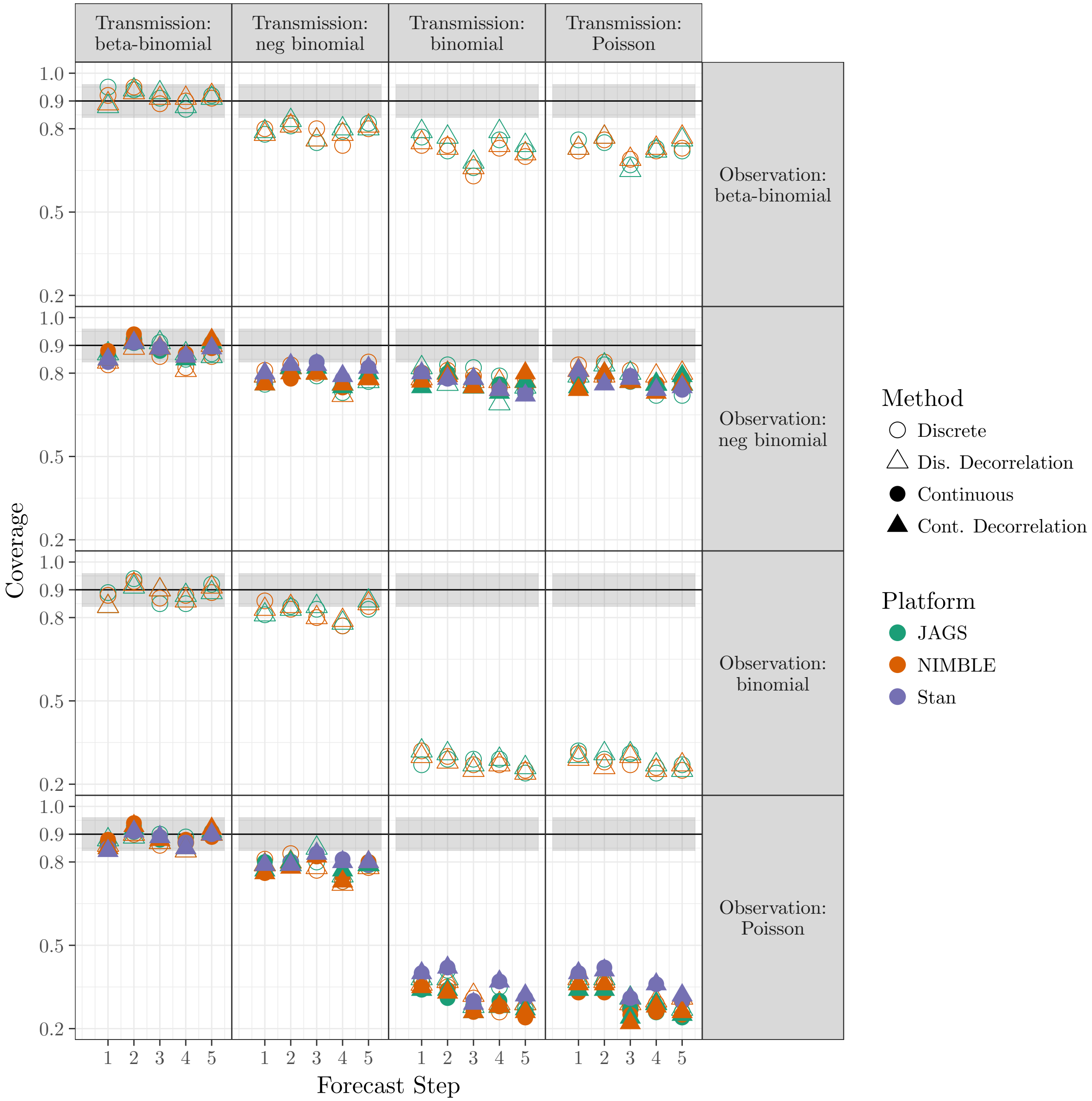

Figure S7. Comparison of coverage for five forecast steps described in Sect. 2.2 across different platforms described in Sect. 2.3.1. See Figure **??** in the main text for details.

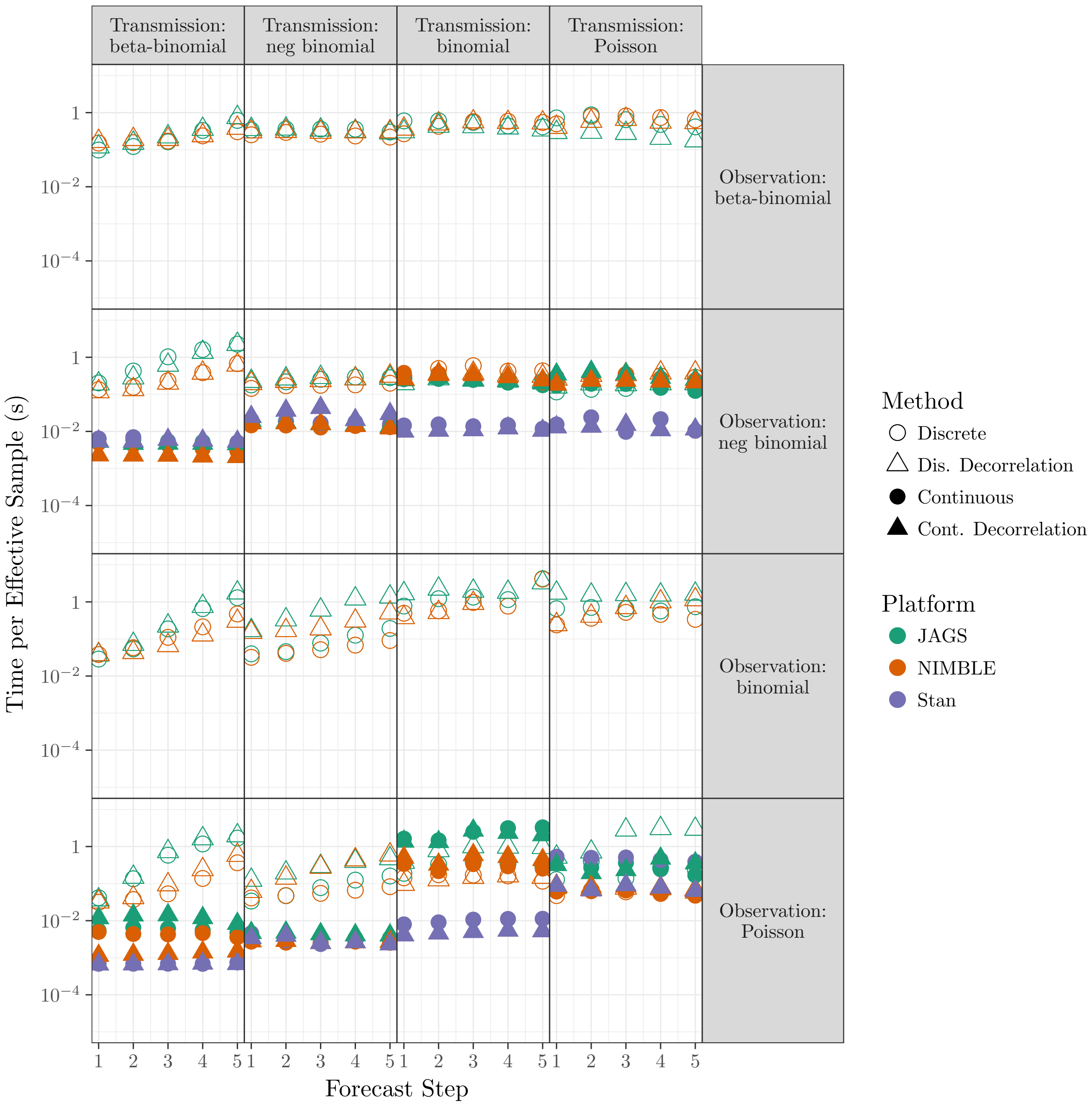

Figure S8. Comparison of sampling efficiency for five forecast steps described in Sect. 2.2 across different platforms described in Sect. 2.3.1. See Figure **??** in the main text for details.

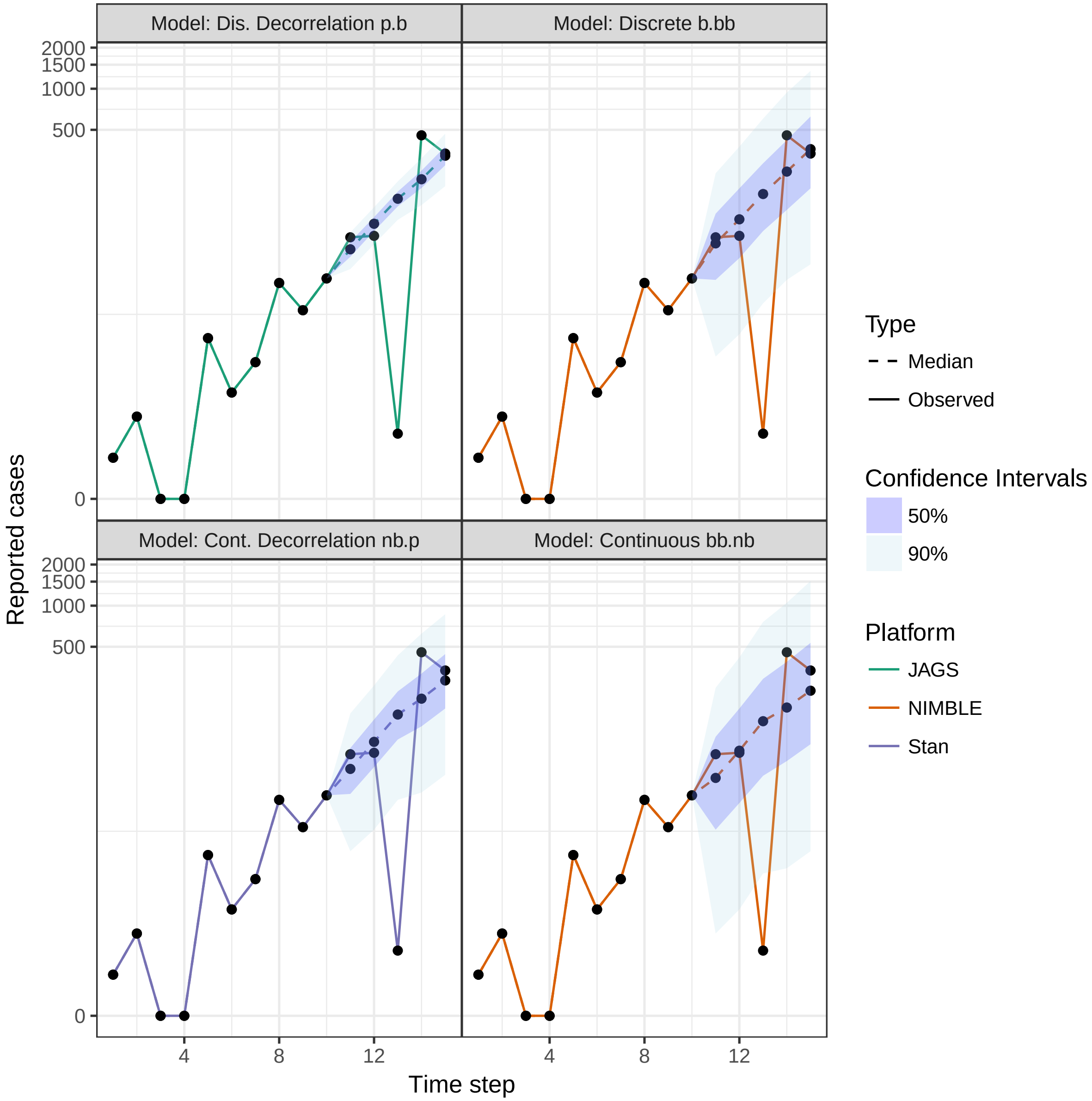

Figure S9. Comparison of forecast using combinations of transmission process, observation process, decorrelation, latent state variables, and platforms described in Sect 2.2 and 2.3.1. Moving from the top to bottom row adds overdispersion in the transmission process (binomial (b) and Poisson (p) to negative-binomial (nb) and beta-binomial (bb)). Moving from left to right adds overdispersion in the observations. Solid line shows the simulated observed cases (15 time steps); dashed line shows the median of the posterior forecast sample with 50% (dark ribbon) and 90% (light ribbon) confidence intervals (last 5 time steps).

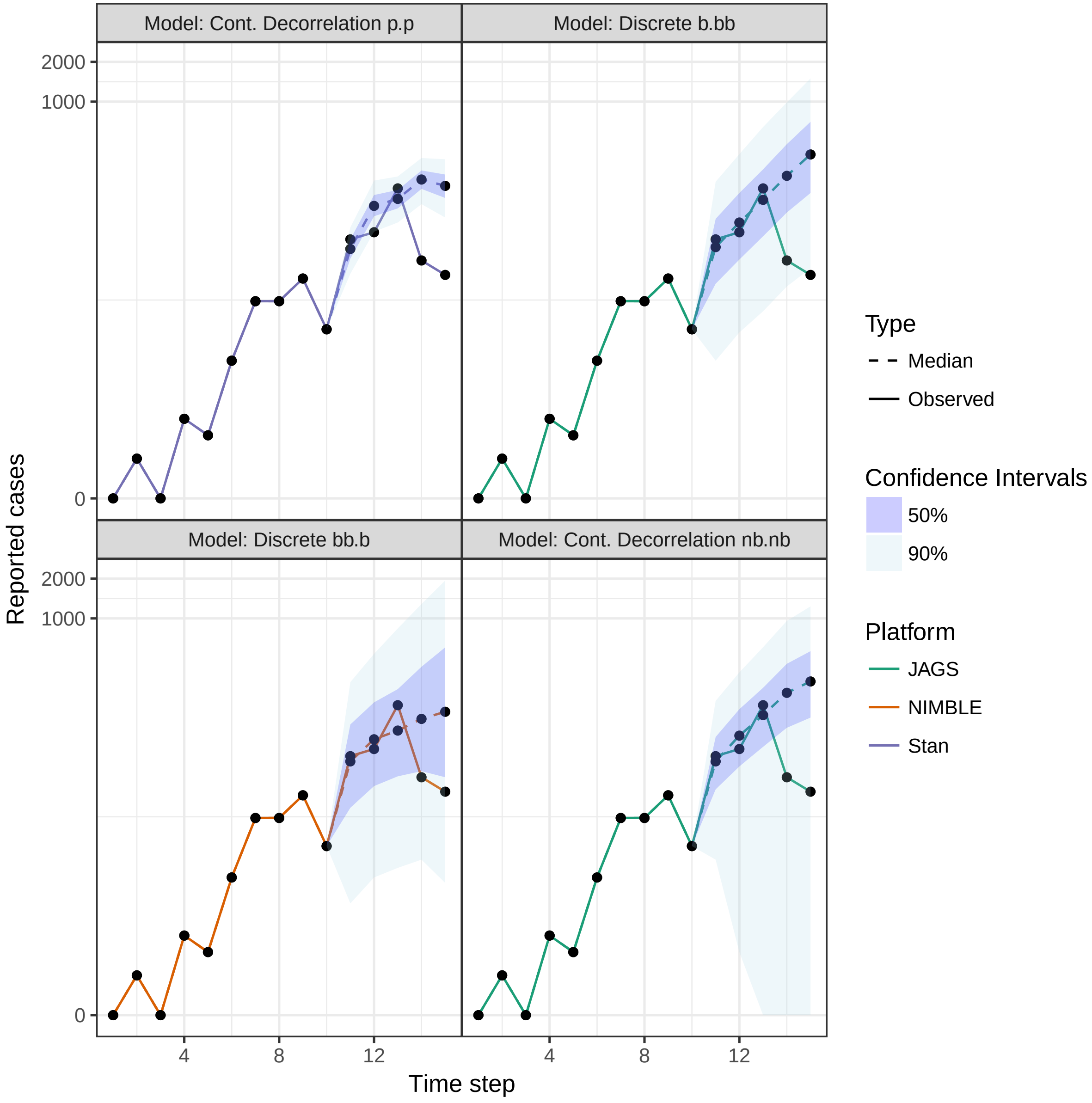

Figure S10. Comparison of forecast using a different set of parameters. See Figure S9 for details.

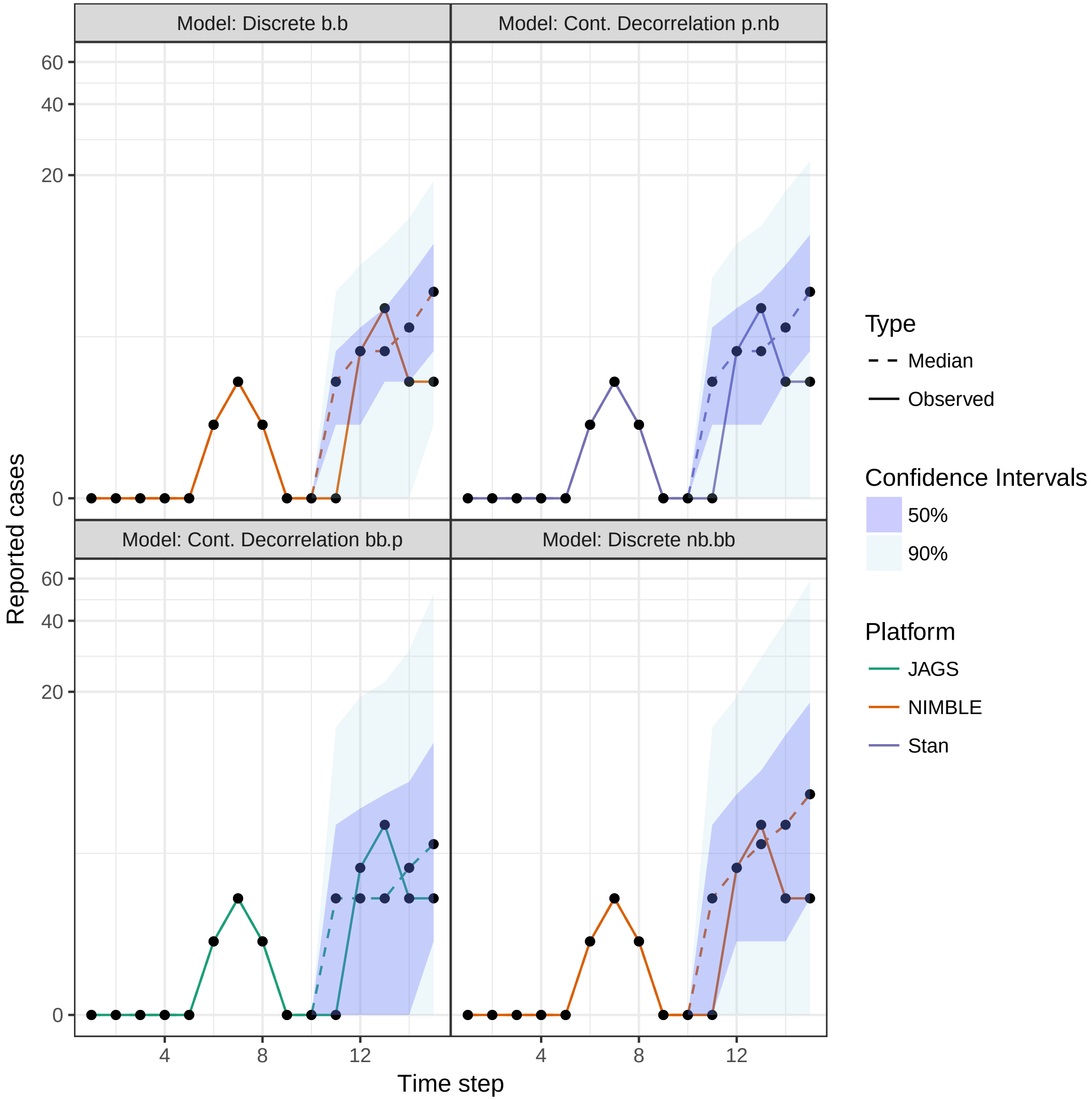

Figure S11. Comparison of forecast of low observed cases. See Figure S9 for details.

Tables

Table 1. Simulation model parameters

Parameter	Description	True	Prior
N	Total population size	Fixed at 100,000	NA
ℓ	Maximum length of the generation interval	Fixed at 5 time steps	NA
$\mathcal{R}_0$	Basic reproductive number	3	Gamma(shape=15,rate=5)
P_{eff}	Effective susceptible proportion of the population	0.5	Beta($\frac{B_{size}}{1-P_{\text{eff}}}, \frac{B_{size}}{P_{\text{eff}}}$)
P_{rep}	Reporting proportion	0.5	Beta($\frac{B_{size}}{1-P_{\text{rep}}}, \frac{B_{size}}{P_{\text{rep}}}$)
G_p	Position parameter for generation interval	0.5	Beta($\frac{2B_{size}}{1-G_p}, \frac{2B_{size}}{G_p}$)
G_s	Shape parameter for generation interval	1	Gamma(shape=5,rate=5)
δ_P	Beta Binomial transmission process dispersion	1	Gamma(shape=10,rate=10)
δ_{obs}	Beta-Binomial Observation process dispersion	1	Gamma(shape=10,rate=10)

Table 2. Fitting model parameters

Parameter	Description	True	Prior
N	Total population size	Fixed at 100,000	NA
ℓ	Maximum length of the generation interval	Fixed at 5 time steps	NA
B_{size}	Beta prior size factor	Fixed at 1	NA
$\mathcal{R}_0$	Basic reproductive number	3	Gamma(shape=15,rate=5)
P_{eff}	Effective susceptible proportion of the population	0.5	Beta($\frac{B_{size}}{1-P_{\text{eff}}}, \frac{B_{size}}{P_{\text{eff}}}$)
P_{rep}	Reporting proportion	0.5	Beta($\frac{B_{size}}{1-P_{\text{rep}}}, \frac{B_{size}}{P_{\text{rep}}}$)
P_{effrep}	Proportion of effective S to I that are observed	$P_{\text{eff}} \times P_{\text{rep}}$	Beta($\frac{B_{size}}{1-P_{\text{effrep}}}, \frac{B_{size}}{P_{\text{effrep}}}$)
ρ	Scale splitting factor	0.5	Beta($\frac{B_{size}}{1-\rho}, \frac{B_{size}}{\rho}$)
G_p	Position parameter for generation interval	0.5	Beta($\frac{2B_{size}}{1-G_p}, \frac{2B_{size}}{G_p}$)
G_s	Shape parameter for generation interval	1	Gamma(shape=5,rate=5)
δ_P	Beta Binomial transmission process dispersion	1	Gamma(shape=10,rate=10)
δ_P (Neg-Binom)	Negative-Binomial Transmission process dispersion	NA	Uniform(min=0,max=100)
δ_{obs}	Beta-Binomial Observation process dispersion	1	Gamma(shape=10,rate=10)
δ_{obs} (Neg-Binom)	Negative-Binomial Transmission process dispersion	NA	Uniform(min=0,max=100)
